# Climate change and substance use disorders – do we understand the risks?

**DOI:** 10.1192/j.eurpsy.2022.2104

**Published:** 2022-09-01

**Authors:** F. Vergunst, H. Berry

**Affiliations:** 1 University of Montreal, Social And Preventive Medicine, Chemin de la Côte Ste-Catherine , Canada; 2 University of Sydney, Faculty Of Medicine And Health, Camperdown, Australia

**Keywords:** Child and adolescent, Psychopathology, Climate change and environment, Addiction

## Abstract

**Introduction:**

Climate change is increasing the frequency and intensity of severe heatwaves, storms, floods, droughts, and wildfires. These events cause widespread economic and social disruption and are undermining population health worldwide. Despite a growing literature on how climate change threatens mental health, its influence on harmful substance use has not been systematically addressed.

**Objectives:**

We propose an explanatory framework explicating the plausible links between climate change-related stressors and an increase in harmful substance use.

**Methods:**

We critically review and synthesise literature documenting the pathways, processes and mechanisms linking climate change to increased substance use vulnerability.

**Results:**

Several plausible pathways link climate change to increased risk of harmful substance use worldwide. These include: (1) anxiety about the impacts of unchecked climate change, (2) destabilisation of psychosocial and economic support systems, (3) increasing rates of mental disorders, and (4) increased physical health burden. Children may face disproportionate risk due to their vulnerability to both mental disorders and substance use, particularly during adolescence. We argue that a developmental life-course perspective situated within a broader ‘systems thinking’ approach provides a coherent framework for understanding how climate change is aggravating the multiple, persistent, interacting risks that influence harmful substance use pathways.

**Conclusions:**

Climate change is already undermining health and wellbeing of global populations. By inference, it is also aggravating pathway to harmful substance use. This is a critical psychosocial problem for individuals and communities alike. Conceptual and methodological work is urgently needed so that effective adaptive and preventive action can be taken.

**Disclosure:**

No significant relationships.

